# Neonatal sepsis in Sana’a city, Yemen: a predominance of *Burkholderia cepacia*

**DOI:** 10.1186/s12879-021-06808-y

**Published:** 2021-10-27

**Authors:** Adeeb Salah, Ibrahim Al-Subol, Ahmed Hudna, Ali Alhaj, Abdulhabib R. Alqubaty, Waleed Farie, Dalal Sulieman, Ola Alnadhari, Talal Alwajeeh, Fawaz Alobathani, Abdullah Almikhlafy, Mohmmed A. K. Mahdy

**Affiliations:** 1 Department of Pathology, Faculty of Medicine and Health Sciences, University of Science and Technology, Sanaa, Yemen; 2 Department of Microbiology, Faculty of Medicine and Health Sciences, University of Science and Technology, Sanaa, Yemen; 3 Department of Pediatrics, Faculty of Medicine and Health Sciences, University of Science and Technology, Sanaa, Yemen; 4 Department of Biochemistry, Faculty of Medicine and Health Sciences, University of Science and Technology, Sanaa, Yemen; 5 Department of Laboratory Medicine, Faculty of Medicine and Health Sciences, University of Science and Technology, Sanaa, Yemen; 6 Department of Pediatrics, C-PLAS Hospital, Sanaa, Yemen; 7 Department of Laboratory Medicine, Faculty of Medicine and Health Sciences, University of Amran, Amran, Yemen; 8 Department of Pediatrics, Al-Kuwait University Hospital, Sanaa, Yemen; 9 Department of Community Medicine, Faculty of Medicine and Health Sciences, University of Science and Technology, Sanaa, Yemen; 10 Department of Parasitology, Faculty of Medicine, University of Sana’a, Sanaa, Yemen; 11 Tropical Disease Research Center, University of Science and Technology, Sanaa, Yemen

**Keywords:** Sepsis, Bacteria, Antibiotic resistance, VITEK II, Blood culture, Gram-negative, *Burkholderia cepacia*

## Abstract

**Background:**

Neonatal sepsis is a global concern with increasing morbidity and mortality. The burden of neonatal sepsis is highest in developing countries, especially in those lacking proper surveillance systems. The causative pathogens and their drug-resistance levels vary between countries with emergence of multidrug resistance organisms. Thus, accurate records on the recent trends of organisms causing neonatal sepsis will provide vital information for appropriate intervention. We aimed to investigate neonatal sepsis, identify its associated factors and causative pathogens and to assess the antibiotic susceptibility patterns in Sana’a city, Yemen.

**Methods:**

A cross-sectional study was conducted on neonates admitted to intensive care units of six hospitals in Sana’a city, Yemen, in the period from January 15, to March 30, 2020. Natal and prenatal medical data were collected using well-structured questionnaire. Neonates were subjected to sepsis work-up including blood culture, complete blood count and C-reactive protein. Organisms were identified by Gram staining and analyzed by the VITEK II system for bacterial bio-typing and antibiotic susceptibility testing.

**Findings:**

Of the 199-neonates with suspected neonatal sepsis, 154 (77.38%) had culture-proven sepsis. Early-onset neonatal sepsis (EOS) was higher (50.25%; 100/199) than late-onset neonatal sepsis (LOS) (27.13%; 54/199). Multivariable analysis identified vaginal delivery as an independent risk factor for neonatal sepsis *p* = 0.005. Majority of isolated bacteria (74.39%) were gram-negative with *Burkholderia cepacia* (39%) and *Klebsiella oxytoca* (13%) being the most common pathogens of EOS and LOS. The most common gram-positive pathogens were *Staphylococcus haemolyticus* (9.1%) and *Staphylococcus epidermidis* (7.1%). *B. cepacia* showed multidrug resistance except for cefepime. All *Klebsiella* species isolates (*100%*) and most *Pantoea* species (*93%*) were ESBL and carbapenemase positive. All *Escherichia coli* and *Acinetobacter baumannii* isolates were ESBL positive. A significant number of gram-positive bacteria showed resistance to vancomycin.

**Conclusion:**

The study findings show a high proportion of neonatal sepsis among neonates admitted to hospitals in Sana’a city with antibiotic-resistant *B. cepacia* being the single most common pathogen causing EOS and LOS. Findings also emphasize the emerging threat of multidrug-resistant bacteria in neonatal units and will help develop evidence-based management of neonatal sepsis in Yemen.

## Background

Neonatal sepsis is a leading cause of neonatal morbidity and mortality worldwide. The global burden of neonatal sepsis and other neonatal infections was estimated at 22 million disability-adjusted life-years (95% UI: 18.9–28.0) [1]. In developing countries, neonatal sepsis is responsible for more than 50% of neonatal deaths. It includes EOS, which occurs within 72 h after birth and LOS [2]. The two types of infections have different clinical manifestations, epidemiology, and pathogens patterns [3–6].

Pathogens causing neonatal sepsis are either acquired from the maternal flora or postnatally from the hospital or community [7]. Bacteria are the most common pathogens, and they vary in the distribution between countries, regions and according to the disease onset EOS versus LOS [7]. The commonly involved bacteria in developing countries include *Klebsiella* species, *Staphylococcus aureus*, *E. coli*, and Group B *Streptococcus* [8, 9]. Recently, *B. cepacia*, a bacterial pathogen that has intrinsic drug resistance and ability to live inside drug products [10, 11], has emerged as a significant pathogen causing neonatal sepsis [12–16].

Yemen is suffering of an ongoing war that started in 2015. The war has destroyed the health system, rendering health facilities either not functioning or partially functioning [17]. There is a paucity of information about neonatal sepsis including its types, causative pathogens patterns and antibiotic susceptibility. Therefore, this study aimed to investigate neonatal sepsis, identify its associated factors and causative pathogens, and to assess the antibiotic susceptibility patterns in Sana’a city, Yemen. Findings from this study will fill gaps of knowledge about neonatal sepsis in Yemen, helping public health authorities to develop an effective control strategy to combat the disease, and guide Yemeni physicians to implement an evidence-based approach for the clinical management of neonatal sepsis.

## Methods

All methods were carried out in accordance with the relevant guidelines and regulations.

### Study design and subjects

This was a cross-sectional study conducted on neonates admitted to neonatal units in six major hospitals in Sana’a city; Al-Kuwait, C-Plas, Al-Gumhory, Al-Sabeen, AL-Thawra, and Family hospitals, in the period from January 15 to March 30, 2020. Neonates who were admitted for at least 72 h with suspected sepsis during the study period were included. Neonates with congenital anomalies and hemolytic jaundice were excluded from the study.

### Diagnosis of neonatal sepsis

Neonatal sepsis was suspected based on the presence of clinical signs or risk factors according to the international recommendations [18] and confirmed as neonatal sepsis if blood culture was positive [18, 19]. Neonatal sepsis was then classified into EOS and LOS [19].

### Questionnaire

Bio and socio-demographic data were collected using a standard questionnaire by face-to-face interviews with the neonate’s mothers. The health condition of the mothers and clinical manifestations of the neonates were collected by physicians using standard tools. Written informed consent was obtained from guardians of all neonates after explaining the study objectives. All procedures described in this study were approved by the Research and Ethics Committee of the Faculty of Medicine and Health Sciences, University of Science and Technology, Sana’a (Approval No. EAU/UST122).

### Laboratory investigations

Laboratory investigations were performed according to standard microbiological techniques [20]. Under aseptic conditions, trained nurses collected blood samples that were used for laboratory investigations of complete blood counts, C-reactive proteins and blood culture. For culture, at least 1 ml of blood was inoculated into BacT/Alert PF plus culture bottle (BIOMERIEUX, France, LOT 4053532) and incubated until the BacT/Alert instrument (BACTEC 9050, Becton Dickinson) signals it either as positive or negative. All positive samples were sub-cultured on choclate agar, blood agar, and MacConkey agar and incubated at 37 °C for 24–48 h. Gram-staining was conducted to differentiate between gram-positive and gram-negative bacteria. A sufficient number of colonies of pure culture was used to suspend the microorganism in 3.0 ml of sterile saline test tube. Pure bacterial suspension was added to bacterial specific identification and sensitivity testing kit device and analyzed by the VITEK II system for bacterial bio-typing and antibiotic susceptibility patterns as instructed in the product information manuals (BIOMERIEUX). VITEK^®^ GN ID identification card (lot 2410933203) was used to characterize gram-negative bacteria and VITEK^®^ GP ID identification card (lot 2420938203) was used to characterize gram-positive bacteria. Different cards were used to perform antibiotic susceptibility tests; AST-GN 87 (lot; 6770912203), AST-GN 72(lot; 5921083103) and AST GN75 (lot; 5951129403) for gram-negative bacteria and AST-GP67 (lot; 1321137103) for gram-positive bacteria. All procedures were conducted for routine diagnostic and therapeutic reasons.

### Statistical analysis

Data were analyzed using IBM SPSS Statistics for Mac, version 23.0 (IBM Corp., Armonk, NY, USA). Categorical variables were presented in frequencies. The association between independent and dependent variables was tested using Pearson’s Chi-squared with reporting odds ratio (OR) and 95% confidence interval (CI). Fisher’s exact test was used when applicable. Multivariable analysis using the binary logistic regression model was conducted, including all variables, and the adjusted OR with its corresponding 95%CI was reported. A *p*-value of < 0.05 was considered significant.

### Ethical consideration

The study protocol was approved by the Research and Ethics Committee of the Faculty of Medicine and Health Sciences, University of Science and Technology, Sana’a, Yemen (Approval No. EAU/UST122).

## Results

### Characteristics of the patients

A total of 199 neonates admitted to referral hospitals in Sana’a city, Yemen, were enrolled in this study. Nearly half of neonates (84, 42.2%) were preterm, majority of them (70, 83.3%) were moderates to late preterm. More than half of neonates (109, 54.8%) were low birth weight. A total of 113 neonates (56.8%) were born by vaginal delivery. The age of 133 neonates (66.8%) was less than 72 h on admission. Tachypnea, lethargy and poor feeding were the most common clinical manifestations observed among neonates (Table 1).

**Table 1:** Characteristics of neonates with suspected sepsis admitted to referral hospitals in Sana’a city, Yemen, in the period from January 15 to March 30, 2020 (N = 199)

Variable	n (%)
Gender	
Female	77 (38.7)
Male	122 (61.3)
Gestational age (weeks)	
≥ 37	115 (57.8)
< 37	84 (42.4)
Mode of delivery	
Cesarean section	86 (43.2)
Vaginal delivery	113 (56.8)
Place of delivery	
Hospital	170 (85.4)
Home	29 (14.6)
Premature rupture of membrane	
No	139 (69.8)
Yes > 18 h	60 (30.2)
Birth weight (g)	
≥ 2500	90 (45.2)
< 2500	109 (54.8)
Age of neonate (h)	
> 72	66 (33.2)
≤ 72	133 (66.8)
Clinical manifestations	
Poor feeding	120 (60.3)
Convulsion	17 (8.5)
Irritability	35 (17.5)
Jaundice	58 (29.1)
Tachypnea	135 (67.8)
Apnea	11 (5.5)
Lethargy	129 (64.8)
Poor sucking	102 (51.2)
Hypothermia	9 (4.5)
Hyperthermia	36 (18.0)

### Culture-confirmed neonatal sepsis and its associated factors

Majority of neonates 77.38% (154/199) had positive blood culture. Of them, 50.25% (100/199) had EOS. Neonates born by vaginal delivery were at three-times higher risk of neonatal sepsis (OR = 3.08, 95% CI 1.54, 6.16; *p* = 0.002) than those whose deliveries were through Cesarean section. Although most neonates born at home acquired neonatal sepsis (OR = 2.8; 95% CI 0.81, 9.87; *p* = 0.09), the significance of association was in the borderline (Table 2). Multivariable analysis using binary logistic regression model identified vaginal delivery as an independent risk factor for neonatal sepsis (adjusted OR = 3.0; 95% CI 1.40, 6.33, *p* = 0.005).

**Table 2:** Factors associated with culture-confirmed neonatal sepsis among septic neonates admitted to referral hospitals in Sana’a city, Yemen, in the period from January 15 to March 30, 2020 (N = 199)

Variable	N	Culture-confirmed neonatal sepsis		
		n (%)	OR (95% CI)	p value
Gender				
Female	77	60 (77.9)	Reference	
Male	122	94 (77)	0.95 (0.48, 1.88)	1.0
Gestational age (weeks)				
≥ 37	115	89 (77.4)	Reference	
< 37	84	65 (77.4)	0.99 (0.51, 1.95)	1.0
Mode of delivery				
Cesarean section	86	57 (66.3)	Reference	
Vaginal delivery	113	97 (85.8)	3.08 (1.54, 6.16)	0.002*
Place of delivery				
Hospital	170	128(75.3)	Reference	
Home	29	26 (89.9)	2.80 (0.81, 9.87)	0.09
Premature rupture of membrane				
No	139	111(79.9)	Reference	
Yes > 18 h	60	43 (71.7)	0.63 (0.31, 1.28)	0.26
Birth weight (g)				
≥ 2500	90	68 (75.6)	Reference	
< 2500	109	86 (78.9)	1.21 (0.62, 2.35)	0.61

N, number of suspected neonates; n, number of culture-confirmed septic neonates; OR, odds ratio; CI, confidence interval

*Was confirmed as independent risk factor using binary logistic regression model (adjusted OR = 3.0; 95% CI 1.40, 6.33, *p* = 0.005)

### Clinical manifestations and hematological factors associated with culture-confirmed sepsis

Culture confirmed neonatal sepsis was significantly associated with hyperthermia (*p* = 0.045) and convulsion (*p* = 0.01). Poor feeding, jaundice, irritability, and lethargy were not significantly associated with neonatal sepsis. Elevated C-reactive protein was significantly associated with neonatal sepsis (*p* = 0.001). One-third (32%) of neonates with proven sepsis had normal white cell counts and two-thirds (59%) had normal platelets counts (Table 3).

**Table 3:** Association of clinical manifestations and hematological factors with culture-confirmed sepsis among septic neonates admitted to referral hospitals in Sana’a city, Yemen, in the period from January 15 to March 30, 2020 (N = 199)

Variable	N	Culture-confirmed neonatal sepsis		
		n (%)	Chi-square value	p value
Clinical manifestations				
Poor feeding				
No	79	58 (73.4)	1.18	0.277
Yes	120	96 (80.0)		
Convulsion^a^				
No	182	137(75.3)		0.015
Yes	17	17 (100)		
Irritability^a^				
No	164	124(75.6)		0.266
Yes	35	30 (85.7)		
Jaundice				
No	141	107(75.9)	0.62	0.430
Yes	58	47 (81.0)		
Lethargy				
No	70	52 (74.3)	0.59	0.411
Yes	129	102(79.1)		
Respiratory rate^a^				
Normal	53	40 (75.5)		0.96
Tachypnea	135	105 (77.8)		
Apnea	11	9 (81.8)		
Temperature^a^				
Normal	154	115 (74.7)		0.045
Hyperthermia	36	33 (91.7)		
Hypothermia	9	6 (66.7)		
Hematological factors				
C-reactive protein				
Non-reactive < 6 ml/dl	74	48 (64.9)	11.3	0.001
Reactive	113	97 (85.8)		
WBC^a^				
Normal	65	49 (75.4)		0.891
High	126	99 (78.6)		
Low	6	5 (83.3)		
Platelets^a^				
Normal	124	91 (73.4)		0.158
High	5	5 (100)		
Low	68	57 (83.8)		

N, number of suspected neonates; n, number of culture-confirmed septic neonates; normal WBC 4000–10,000/mm^3^; normal platelets 150,000–450,000; normal respiratory rate, 30–50permint

^a^Fisher’s exact test was used

### Pathogens causing neonatal sepsis

Table 4 represents the distribution of pathogens causing neonatal sepsis. Out of the 154 neonates with culture-confirmed sepsis, 152 were infected by bacteria, while two were infected by *Candida albicans.* Of the 152 neonates with proven bacterial culture, nine had mixed bacterial growth. We isolated 161 bacterial pathogens, 119 (74%) were gram-negative while 42 (26%) were gram-positive*. B. cepacia* (37%) was the most common organism causing neonatal sepsis with higher prevalence among EOS (38%) than LOS (35%), followed by *K. oxytoca* (11.6%), which was higher among LOS (14.8%) than EOS (10.0%). *Pantoea agglomerans* caused neonatal sepsis in nine neonates (5.8%). The main gram-positive pathogens were *S. haemolyticus* (9.1%), *S. epidermidis* (7.1%) and *Staphylococcus hominis* (5.1%). Coagulase-negative Staphylococci (CONS) was the most common gram-positive organisms. *S. haemolyticus* emerged as the most frequently CONS.

**Table 4:** Organisms causing neonatal sepsis among neonates admitted to referral hospitals in Sana’a city, Yemen, in the period from January 15 to March 30, 2020 (N = 154)

Isolated organism	Proportion of isolated pathogen n (%)		
	Early onset^a^ sepsis (N = 100)	Late onset sepsis (N = 54)	Total (N = 154)
Single infection			
* Burkholderia cepacia*	38 (38.0)	19 (35.2)	57 (37.0)
* Klebsiella oxytoca*	10 (10.0)	8 (14.8)	18 (11.6)
* Pantoea agglomerans*	7 (7.0)	2 (3.7)	9 (5.8)
* Pseudomonas aeruginosa*	4 (4.0)	2 (3.7)	6 (3.8)
* Klebsiella pneumoniae*	2 (2.0)	2 (3.7)	4 (2.5)
* Pantoea dispersa*	3 (3.0)	1 (1.8)	4 (2.5)
* Acinetobacter baumannii*	2 (2.0)	0 (0)	2 (1.3)
* Acinetobacter lwoffii*	2 (2.0)	1 (1.8)	3 (1.9)
* Enterobacter cloacae complex*	0 (0)	1 (1.8)	1 (0.64)
* Escherichia coli*	1 (1.0)	1 (1.8)	2 (1.3)
* Achromobacter denitrificans*	0 (0)	1 (1.8)	1 (0.64)
* Sphingomonas paucimobilis*	1 (1.0)	0 (0)	1(0.64)
* Staphylococcus haemolyticus*	8 (8.0)	4 (7.4)	12 (7.8)
* Staphylococcus epidermidis*	6 (6.0)	5 (9.2)	11 (7.1)
* Staphylococcus hominis*	4 (4.0)	2 (3.7)	6 (3.8)
* Staphylococcus aureus*	2 (2.0)	1 (1.8)	3 (1.9)
* Staphylococcus saprophyticus*	2 (2.0)	0 (0)	2 (1.3)
* Enterococcus faecalis*	0 (0)	1 (1.8)	1 (0.64)
* Candida albicans*	2 (2.0)	0 (0)	2 (1.3)
Double infection			
* B. cepacia and S. hominis*	2&2(2.0)	0 (0)	2 (1.3)
* B. cepacia and S. haemolyticus*	0 (0)	1&1 (1.8)	1 (0.64)
* K. oxytoca and Serratia marcescens*	1&1(1.0)	0 (0)	1 (0.64)
* P. agglomerans and K. oxytoca*	0 (0.0)	1&1 (1.8)	1 (0.64)
* K. pneumoniae and E. cloacae complex*	1&1 (1.0)	0 (0.0)	1 (0.64)
* A. baumannii and S. saprophyticus*	1&1 (1.0)	0 (0.0)	1 (0.64)
* E. cloacae complex and E. faecalis*	0 (0)	1&1 (1.8)	1 (0.64)
* S. haemolyticus* and *Streptococcus. agalactiae*	1&1 (1.0)	0 (0.0)	1 (0.64)
Total number of isolated organisms	106	57	163

N, number of patients

^a^Early onset sepsis ≤ 72 h and Late onset sepsis > 72 h

### Antibiotic susceptibility

Most isolated pathogens showed antimicrobial resistance (AMR) to the commonly used antibiotics (ampicillin, gentamicin, amikacin), cephalosporins and carbapenems (Tables 5 and 6). *B. cepacia*, the most common pathogen caused neonatal sepsis in this study, was highly resistant to ampicillin/sulbactam, gentamicin, tobramycin, tetracycline, amoxicillin/clavulanic acid, cefalotin, cefazolin, cefuroxime, cefoxitin, cefpodoxime, imipenem, and amikacin*.* However, it was 100% susceptible to cefepime. *K. oxytoca* was susceptible to gentamicin, ciprofloxacin, levofloxacin, tetracycline, nitrofurantoin, and trimethoprim/sulfamethoxazole. *Klebsiella* species (100%) and *Pantoea* species (*93%*) were carbapenemase positive and ESBL positive. All isolates of *E. coli* and *A. baumannii* were ESBL positive (Table 5). A significant number of gram-positive isolates were resistant to vancomycin. Most gram-positive bacteria were cefoxitin screening positive and sensitive to moxifloxacin, linezolid and rifampicin. *S. haemolyticus* isolates were resistant to ampicillin and gentamicin (Table 6). *Staphylococcus* species showed resistance to fluoroquinolones (ciprofloxacin).

**Table 5:** Antibiotic resistance levels of gam-negative bacteria isolated from septic neonates admitted to referral hospitals in Sana’a city, Yemen, in the period from January 15 to March 30, 2020

Pathogen	Ampicillin sulbactam		Ampicillin		Amo/CA		Piperacillin/tazobactam		Cefalotin		Cefazolin		Cefuroxime	
	N	%	N	%	N	%	N	%	N	%	N	%	N	%
*B. cepacia*	54	100	60	100	28	100	60	15	27	100	60	100	28	100
*K. oxytoca*	20	100	20	100	3	100	20	100	3	100	20	100	3	100
*P. agglomerans*	NA	NA	NA	NA	9	100	9	100	9	100	10	100	9	100
*P. aeruginosa*	6	100	6	100	4	100	6	0	4	100	6	100	4	75
*K. pneumoniae*	5	100	5	100	1	100	5	100	1	100	5	100	1	100
*P. dispersa*	NA	NA	NA	NA	NA	NA	4	100	NA	NA	4	75	NA	NA
*A. baumannii*	3	33	1	100	3	33	3	33	1	100	3	100	1	100
*A. lwoffii*	3	100	3	100	2	100	3	33	3	100	3	100	2	100
*E. cloacae complex*	NA	NA	NA	NA	1	100	3	33	1	100	3	100	1	100
*E. coli*	2	50	2	100	NA	NA	2	100	NA	NA	2	100	NA	NA
*A. denitrificans*	1	0	1	100	1	0	1	0	1	100	1	100	1	100
*S. marcescens*	NA	NA	NA	NA	NA	NA	1	100	NA	NA	1	100	NA	NA
*S. paucimobilis*	1	100	1	100	NA	NA	1	100	NA	NA	1	100	NA	NA

Pathogen	Cefuroxime axetil		Cefoxitin		Cefpodoxime		Ceftazidime		Ceftriaxone		Cefepime	
	N	%	N	%	N	%	N	%	N	%	N	%
*B. cepacia*	28	100	59	100	28	100	60	3.3	60	10	60	0
*K. oxytoca*	3	100	20	100	3	100	20	100	20	100	20	100
*P. agglomerans*	9	100	10	100	9	100	10	100	10	100	10	100
*P.aeruginosa*	4	100	6	100	4	75	6	0	6	66	6	0
*K. pneumoniae*	1	100	5	80	1	100	5	100	5	100	5	100
*P. dispersa*	NA	NA	4	75	NA	NA	4	75	4	75	4	75
*A. baumannii*	1	100	3	100	1	100	3	100	3	100	3	33
*A. lwoffii*	2	100	3	100	2	100	3	33	3	0	3	0
*E. cloacae complex*	1	100	3	100	1	100	3	33	3	33	3	33
*E. coli*	NA	NA	2	50	NA	NA	2	100	2	100	2	100
*A. denitrificans*	1	100	1	100	1	100	1	0	1	100	1	0
*S. marcescens*	NA	NA	1	0	NA	NA	1	100	1	100	1	100
*S. paucimobilis*	NA	NA	1	100	NA	NA	1	0	1	100	1	0

Pathogen	Gentamicin		Tobramycin		Ciprofloxacin		Levofloxacin		Tetracycline		FT		SXT	
	N	%	N	%)	N	%	N	%	N	%	N	%	N	%
*B. cepacia*	59	100	60	100	60	98	60	80	28	100	60	100	60	5
*K. oxytoca*	20	0	20	100	20	0	20	0	3	0	20	0	20	0
*P. agglomerans*	10	100	10	100	10	0	10	0	9	0	10	100	10	0
*P. aeruginosa*	6	66	6	66	6	17	6	33	4	100	6	100	6	66
*K. pneumoniae*	5	20	5	100	5	0	5	0	1	0	5	20	5	40
*P. dispersa*	4	100	4	100	4	25	4	0	NA	NA	4	75	3	33
*A. baumannii*	3	66	3	66	3	33	3	33	1	0	3	100	3	100
*A. lwoffii*	3	100	3	100	3	0	3	66	2	100	3	100	3	66
*E. cloacae complex*	3	33	3	33	3	33	3	0	1	0	3	33	3	33
*E. coli*	2	0	2	50	2	50	2	50	NA	NA	2	0	2	0
*A. denitrificans*	1	100	1	100	1	0	1	0	1	0	1	100	1	0
*S. marcescens*	1	100	1	100	1	0	1	0	NA	NA	1	100	1	0
*S. paucimobilis*	1	100	1	100	1	0	1	100	NA	NA	1	100	1	0

Pathogen	Imipenem		Ertapenem		Meropenem		Amikacin		ESBL		Carbapenemase	
	N	%	N	%	N	%	N	%	N	%	N	%
*B. cepacia*	7	100	NA	NA	53	13	53	100	–	–	NA	NA
*K. oxytoca*	1	100	20	100	20	100	20	65	20	100	20	100
*P. agglomerans*	7	100	8	100	10	100	10	100	10	100	10	100
*P. aeruginosa*	2	100	NA	NA	6	0	6	66		–	–	–
*K. pneumoniae*	NA	NA	5	80	5	80	5	40	5	100	5	80
*P. dispersa*	NA	NA	4	50	4	50	4	25	4	75	4	50
*A. baumannii*	1	0	NA	NA	NA	NA	NA	NA	3	100	NA	NA
*A. lwoffii*	NA	NA	NA	NA	1	100	3	100		–	–	–
*E. cloacae complex*	NA	NA	3	33	3	33	3	33	3	33	1	Pos
*E. coli*	NA	NA	2	0	2	0	2	0	2	100	–	–
*A. denitrificans*	1	0	NA	NA	1	0	1	100	1	Pos	–	–
*S. marcescens*	NA	NA	1	0	1	0	1	0	1	Pos	–	–
*S. paucimobilis*	NA	NA	NA	NA	1	100	1	100		–	Pos	Pos

N: the number of isolated bacteria tested for antibiotic sensitivity; %: the percentage of resistant bacteria; NA: not analyzed; pos: positive; Amo/CA: amoxicillin/clavulanic acid; FT: nitrofurantoin; SXT: trimethoprim/sulfamethoxazole; ESBLPos: positive, ESBL-positive, resistance for all penicillins, cephalosporins and aztreonam

**Table 6:** Antibiotic resistance levels of gam-positive bacteria isolated from septic neonates admitted to referral hospitals in Sana’a city, Yemen, in the period from January 15 to March 30, 2020

Pathogen	Cefoxitin screen, positivity		Benzylpenicillin		Ampicillin		Oxacillin		Gentamicin high level		Streptomycin high level		Gentamicin		Ciprofloxacin		Levofloxacin		Moxifloxacin		Inducible clindamycin resistance	
	N	%	N	%	N	%	N	%	N	%	N	%	N	%	N	%	N	%	N	%	N	%
*S. haemolyticus*	10	100	NA	NA	4	100	NA	NA	NA	NA	NA	NA	13	85	10	90	10	90	10	50	10	20
*S. epidermidis*	11	63	NA	NA	NA	NA	NA	NA	NA	NA	NA	NA	11	9.0	11	9	11	9	11	0	11	27.3
*S. hominis*	8	87.5	NA	NA	NA	NA	NA	NA	NA	NA	NA	NA	8	37.5	8	25	8	25	8	0	8	50
*S. aureus*	3	66.6	NA	NA	NA	NA	NA	NA	NA	NA	NA	NA	3	0	3	66.6	3	66	3	0	3	33.3
*E. faecalis*	NA	NA	NA	NA	NA	NA	NA	NA	2	0	2	0	NA	NA	2	0	2	0	NA	NA	NA	NA
*S. saprophyticus*	3	0	NA	NA	NA	NA	NA	NA	NA	NA	NA	NA	3	0	3	0	3	0	3	0	3	66.6
*S. agalactiae*	NA	NA	NA	NA	NA	NA	NA	NA	NA	NA	NA	NA	NA	NA	NA	NA	1	S	NA	NA	NA	NA

	Erythromycin		Clindamycin		Quinupristin/dalfopristin		Linezolid		vancomycin		Tetracycline		Tigecycline		Nitrofurantoin		Rifampicin		Trimethoprim/sulfamethoxazole	
	N	%	N	%	N	%	N	%	N	%	N	%	N	%	N	%	N	%	N	%
*S. haemolyticus*	14	92.8	14	28.5	10	0	14	0	14	28.5	10	10	10	0	10	0	10	10	14	43
*S. epidermidis*	11	36.3	11	36.3	11	9	11	9	11	36.3	11	45.4	11	0	11	0	11	9	11	54.5
*S. hominis*	8	75	8	50	8	0	8	0	8	25	8	37.5	8	0	8	12.5	8	12.5	8	25
*S. aureus*	3	66	3	66.6	3	0	3	0	3	33.6	3	33.3	2	0	3	0	3	0	3	0
*E. faecalis*	2	100	NA	NA	2	100	2	0	2	50	2	50	2	0	2	0	NA	NA	NA	NA
*S. saprophyticus*	3	100	3	66.6	3	0	3	0	3	66.6	3	0	3	0	3	33.3	3	0	3	0
*S. agalactiae*	NA	NA	1	0	1	0	1	0	1	0	1	100	1	100	NA	NA	NA	NA	NA	NA

N: the number of isolated bacteria tested for antibiotic sensitivity, %: the percentage of resistant bacteria, NA: not analyzed

## Discussion

In this multicentric cross-sectional study we report a high proportion of culture-confirmed neonatal sepsis, accounting for two-thirds (77.38%) of admitted neonates in the referral hospitals in Sana’a city in Yemen. EOS was more common than LOS and *B. cepacia* emerged as the predominant causative organism of both EOS and LOS. Majority of the isolated pathogens were resistant to commonly used antibiotics.

Culture confirmed sepsis was high in our study. However, the proportion of culture-confirmed neonatal sepsis varied between studies from developing countries; 62.8% in Pakistan [21], 57% in Yemen [22], 45.9% in Egypt [23], 44.7% in Ethiopia [24], 24% in Tanzania [25] and 12.6% in Nepal [26]. The differences in the proportion of neonatal sepsis between countries may be due to several factors including sample size and the different used techniques.

In this study, EOS was about two times higher than LOS, which is consistent with other reports from developing countries [22, 23, 26, 27]. However, it is inconsistent with reports from developed countries where LOS is the predominant type of neonatal sepsis [28, 29]. The predominance of EOS in developing countries can be attributed to low quality health services and poor hygiene. This is mainly due to illiteracy, ignorance, cultural beliefs, and prejudices [19]. Infection may be of the maternal genital tract or from delivery rooms or neonatal units [19, 30].

Similar to previous studies neonates born by vaginal delivery were at a higher risk of neonatal sepsis compared with those delivered through Cesarean section [23, 27]. Further, culture confirmed sepsis was high (89.9%) among home delivered neonates. Conversely, majority (75.3%) of the hospital delivered neonates had culture confirmed sepsis. These results emphasize on both the vertical and nosocomial transmission of the causative pathogens.

In this study, gram-negative bacteria were the most common cause of EOS and LOS, which is consistent with previous reports from developing countries [8, 9, 22, 31]. Unexpectedly, *B. cepacia* emerged as the most common cause of neonatal sepsis. It was found in all neonatal units. Moreover, it was resistant to aminoglycosides, fluoroquinolones and imipenem. *B. cepacia* has been reported to cause multiple hospital outbreaks and significant neonatal septicemia in different countries [12–16]. Also, it has been isolated from intravenous solutions, mouthwash, disinfectant and medical devices [31–33]. This organism is often overlooked and reported as *Pseudomonas* species [33, 34]. To our knowledge, this is the first report of *B. cepacia* from Yemen.

*Staphylococcus haemolyticus* was the most frequently isolated CONS in this study. Most isolated *S. haemolyticus* showed variable degrees of antibiotic resistance, which is the nature of *S. haemolyticus* to acquire antibiotic resistance due to unusual genome plasticity [35]. So, this organism has a great ability to survive in the hospital environment, especially on medical devices.

Our results of antimicrobial resistance profile are consistent with other findings. Though some of the previous reports were based on cultures from different kinds of medical specimens and performed by conventional methods [8, 24, 28, 36–39]. Most of the isolated gram-negative and gram-positive pathogens were resistant to commonly used antibiotics, penicillins, cephalosporins and carbapenems. Increased antimicrobial resistance in developing countries is due to multiple factors including poverty, self-medication, unregulated supply and drug smuggling, misguided practice and inappropriate prescriptions [40, 41]. Such factors may provoke changes in causative agents together with their change in antibiotic susceptibility patterns [40, 41]. Markedly, majority of neonates in this study received antibiotics before sampling. The presence of ESBL and carbapenemase-positive gram-negative bacteria is an alarming sign both locally and globally and increases the burden of neonatal sepsis in Yemen.

Proven sepsis was associated with elevated CRP. Elevated CRP indicates activation of the immune system and is commonly used as an indicator of bacterial sepsis [18, 19]. The variation in the total white cell counts and platelet counts is considered a hematological response to inflammation, but there was no association between the culture-positive and culture-negative groups regarding white cell or platelets counts. This may be explained by considering evolving neonatal immune system [18, 19].

## Conclusions

Culture-positive neonatal sepsis is high in Sana’a city with EOS representing two-thirds of the cases. *B. cepacia* followed by *K. oxytoca* and *S. haemolyticus* were the most common causes of both EOS and LOS. Majority of the isolated bacterial pathogens showed a high level of resistance to commonly used antibiotics. Vaginal delivery held high risk for developing neonatal sepsis. The study results emphasize the emergence of multidrug-resistant bacteria in the NICUs and might serve as a baseline for proper medical treatment of neonatal sepsis in Sana’a city.

## Data Availability

The datasets used and analyzed during the current study are available from the corresponding author on reasonable request.

## Abbreviations

CRP: C-reactive protein
ESBL: Extended-Spectrum β-lactamase
EOS: Early-onset sepsis
LOS: Late-onset sepsis

## References

[1] DALYs GBD, Collaborators H (2018). Global, regional, and national disability-adjusted life-years (DALYs) for 359 diseases and injuries and healthy life expectancy (HALE) for 195 countries and territories, 1990–2017: a systematic analysis for the Global Burden of Disease Study 2017. Lancet.

[2] Simonsen KA, Anderson-Berry AL, Delair SF, Davies HD (2014). Early-onset neonatal sepsis. Clin Microbiol Rev.

[3] Kristóf K, Kocsis E, Nagy K (2009). Clinical microbiology of early-onset and late-onset neonatal sepsis, particularly among preterm babies. Acta Microbiol Immunol Hung.

[4] Dong Y, Speer CP (2015). Late-onset neonatal sepsis: recent developments. Arch Dis Child Fetal Neonatal Ed.

[5] Afonso EDP, Blot S (2017). Effect of gestational age on the epidemiology of late-onset sepsis in neonatal intensive care units—a review. Expert Rev Anti Infect Ther.

[6] You T, Zhang H, Guo L, Ling K-R, Hu X-Y, Li L-Q (2020). Differences in clinical characteristics of early- and late-onset neonatal sepsis caused by Klebsiella pneumoniae. Int J Immunopathol Pharmacol.

[7] Shane AL, Sánchez PJ, Stoll BJ (2017). Neonatal sepsis. Lancet.

[8] Downie L, Armiento R, Subhi R, Kelly J, Clifford V, Duke T (2013). Community-acquired neonatal and infant sepsis in developing countries: efficacy of WHO’s currently recommended antibiotics–systematic review and meta-analysis. Arch Dis Child.

[9] Sgro M, Campbell DM, Mellor KL, Hollamby K, Bodani J, Shah PS (2020). Early-onset neonatal sepsis: organism patterns between 2009 and 2014. Paediatr Child Health.

[10] Singhal T, Shah S, Naik R (2015). Outbreak of Burkholderia cepacia complex bacteremia in a chemotherapy day care unit due to intrinsic contamination of an antiemetic drug. Indian J Med Microbiol.

[11] Glowicz J, Crist M, Gould C, Moulton-Meissner H, Noble-Wang J, de Man TJB (2018). A multistate investigation of health care-associated *Burkholderia cepacia* complex infections related to liquid docusate sodium contamination, January–October 2016. Am J Infect Control.

[12] Mali S, Dash L, Gautam V, Shastri J, Kumar S (2017). An outbreak of *Burkholderia cepacia* complex in the paediatric unit of a tertiary care hospital. Indian J Med Microbiol.

[13] Antony B, Cherian EV, Boloor R, Shenoy KV (2016). A sporadic outbreak of *Burkholderia cepacia* complex bacteremia in pediatric intensive care unit of a tertiary care hospital in coastal Karnataka, South India. Indian J Pathol Microbiol.

[14] Sundaram V, Kumar P, Dutta S, Mukhopadhyay K, Ray P, Gautam V (2009). Blood culture confirmed bacterial sepsis in neonates in a North Indian tertiary care center: changes over the last decade. Jpn J Infect Dis.

[15] Bharara T, Chakravarti A, Sharma M, Agarwal P (2020). Investigation of *Burkholderia cepacia* complex bacteremia outbreak in a neonatal intensive care unit: a case series. J Med Case Rep.

[16] Yonas E, Damay V, Pranata R, Nusarintowati N (2018). Infective endocarditis due to *Burkholderia cepacia* in a neonate: a case report. J Med Case Rep.

[17] Qirbi N, Ismail SA (2017). Health system functionality in a low-income country in the midst of conflict: the case of Yemen. Health Policy Plan.

[18] Kliegman RM, Stanton BF, St. Geme JW, Nina F, Lielgman RM, Behrman RE, Jenson H, Staton B (2016). Nelson text book of pediatrics. Sepsis septic shock and systemic inflammatory response syndrome.

[19] Zea-Vera A, Ochoa TJ (2015). Challenges in the diagnosis and management of neonatal sepsis. J Trop Pediatr.

[20] Isenbergh HD (2004). Clinical microbiology procedures handbook.

[21] Rahman S, Hameed A, Roghani MT, Ullah Z (2002). Multidrug resistant neonatal sepsis in Peshawar, Pakistan. Arch Dis Child Fetal Neonatal Ed.

[22] Al-Shamahy HA, Sabrah AA, Al-Robasi AB, Naser SM (2012). Types of bacteria associated with neonatal sepsis in Al-Thawra University Hospital, Sana’a, Yemen, and their antimicrobial profile. Sultan Qaboos Univ Med J.

[23] Shehab El-Din EMR, El-Sokkary MMA, Bassiouny MR, Hassan R (2015). Epidemiology of neonatal sepsis and implicated pathogens: a study from Egypt. Biomed Res Int.

[24] Shitaye D, Asrat D, Woldeamanuel Y, Worku B (2010). Risk factors and etiology of neonatal sepsis in Tikur Anbessa University Hospital, Ethiopia. Ethiop Med J.

[25] Mhada TV, Fredrick F, Matee MI, Massawe A (2012). Neonatal sepsis at Muhimbili National Hospital, Dar es Salaam, Tanzania; aetiology, antimicrobial sensitivity pattern and clinical outcome. BMC Public Health.

[26] Ansari S, Nepal HP, Gautam R, Shrestha S, Neopane P, Chapagain ML (2015). Neonatal septicemia in Nepal: early-onset versus late-onset. Int J Pediatr.

[27] Kamath S, Mallaya S, Shenoy S (2010). Nosocomial infections in neonatal intensive care units: profile, risk factor assessment and antibiogram. Indian J Pediatr.

[28] Vergnano S, Menson E, Kennea N, Embleton N, Russell AB, Watts T (2011). Neonatal infections in England: the NeonIN surveillance network. Arch Dis Child Fetal Neonatal Ed.

[29] Stoll BJ, Hansen NI, Sánchez PJ, Faix RG, Poindexter BB, Van Meurs KP (2011). Early onset neonatal sepsis: the burden of group B Streptococcal and *E. coli* disease continues. Pediatrics.

[30] Zaidi AKM, Thaver D, Ali SA, Khan TA (2009). Pathogens associated with sepsis in newborns and young infants in developing countries. Pediatr Infect Dis J.

[31] Dizbay M, Tunccan OG, Sezer BE, Aktas F, Arman D (2009). Nosocomial Burkholderia cepacia infections in a Turkish university hospital: a five-year surveillance. J Infect Dev Ctries.

[32] Doit C, Loukil C, Simon A-M, Ferroni A, Fontan J-E, Bonacorsi S (2004). Outbreak of *Burkholderia**cepacia* bacteremia in a pediatric hospital due to contamination of lipid emulsion stoppers. J Clin Microbiol.

[33] Sousa SA, Ramos CG, Leitão JH (2011). *Burkholderia cepacia* complex: emerging multihost pathogens equipped with a wide range of virulence factors and determinants. Int J Microbiol.

[34] Devanga Ragupathi NK, Veeraraghavan B (2019). Accurate identification and epidemiological characterization of *Burkholderia cepacia* complex: an update. Ann Clin Microbiol Antimicrob.

[35] Czekaj T, Ciszewski M, Szewczyk EM (2015). Staphylococcus haemolyticus—an emerging threat in the twilight of the antibiotics age. Microbiology (Reading).

[36] Badulla WFS, Alshakka M, Mohamed Ibrahim MI (2020). Antimicrobial resistance profiles for different isolates in Aden, Yemen: a cross-sectional study in a resource-poor setting. Biomed Res Int.

[37] Dagnew M, Yismaw G, Gizachew M, Gadisa A, Abebe T, Tadesse T (2013). Bacterial profile and antimicrobial susceptibility pattern in septicemia suspected patients attending Gondar University Hospital, Northwest Ethiopia. BMC Res Notes.

[38] Awad HA, Mohamed MH, Badran NF, Mohsen M, Abd-Elrhman A-SA (2016). Multidrug-resistant organisms in neonatal sepsis in two tertiary neonatal ICUs, Egypt. J Egypt Public Health Assoc.

[39] Viswanathan R, Singh AK, Basu S, Chatterjee S, Sardar S, Isaacs D (2012). Multi-drug resistant gram negative bacilli causing early neonatal sepsis in India. Arch Dis Child Fetal Neonatal Ed.

[40] Chokshi A, Sifri Z, Cennimo D, Horng H (2019). Global contributors to antibiotic resistance. J Glob Infect Dis.

[41] Ayukekbong JA, Ntemgwa M, Atabe AN (2017). The threat of antimicrobial resistance in developing countries: causes and control strategies. Antimicrob Resist Infect Control.

